# Delayed-Onset Immune-Mediated Hepatitis Following Pembrolizumab Discontinuation: A Case Report

**DOI:** 10.7759/cureus.93926

**Published:** 2025-10-06

**Authors:** Connor Barry, Jialing Huang, Chukwunonso B Ubanatu, Joshua Bozym

**Affiliations:** 1 Internal Medicine, Geisinger Health System, Wilkes Barre, USA; 2 Pathology, Geisinger Danville Hospital, Wilkes Barre, USA; 3 Public Health, Drexel Dornsife School of Public Health, Philadelphia, USA; 4 Medicine, Geisinger Health System, Wilkes Barre, USA

**Keywords:** delayed onset hepatitis, drug-induced hepatitis, immune checkpoint inhibitor, immune-checkpoint inhibitor adverse effects, immune related adverse events (iraes), immunotherapy, keytruda, late onset immune hepatitis, pembrolizumab

## Abstract

Immune checkpoint inhibitors (ICIs) such as pembrolizumab (Keytruda) have transformed the treatment landscape for various malignancies, yet they are associated with immune-related adverse events (irAEs), including hepatotoxicity. While most cases of ICI-induced hepatitis occur within weeks of initiating therapy, delayed-onset presentations are increasingly recognized. We report a case of severe transaminase elevation developing three months after cessation of pembrolizumab in a patient with a history of gallbladder carcinoma. Her chemotherapy was placed on hold after a recent stroke led to a worsening functional status, with the initial intent to resume pembrolizumab once her functional status normalized. Workup excluded infectious, metabolic, and autoimmune causes. Liver biopsy revealed immune-mediated hepatitis with histologic evidence of only mild cholestasis. The patient responded to corticosteroid therapy, supporting the diagnosis of ICI-induced hepatotoxicity. This case underscores the importance of maintaining a high index of suspicion for immune-mediated liver injury well beyond the period of active immunotherapy and highlights the diagnostic value of liver biopsy in complex presentations.

## Introduction

Pembrolizumab is a programmed cell death protein 1 (PD-1) immune checkpoint inhibitor (ICI) widely used in the treatment of metastatic and locally advanced malignancies. By inhibiting PD-1, it reactivates cytotoxic T lymphocytes to target tumor cells. However, this immune reactivation may also result in unintended immune-related adverse events (irAEs), including hepatotoxicity. The occurrence of irAEs depends on the organ involved as well as the drug and tumor types [1]. Most ICI-related hepatotoxic effects occur within a few weeks to a month of initiating therapy, but cases with delayed onset are increasingly reported, with clinical abnormalities sometimes manifesting months after cessation of therapy. Most of these effects are reversible, except for endocrine-related abnormalities [2]. Immune-mediated liver injury can present with hepatocellular, cholestatic, or mixed injury patterns and may clinically and histologically resemble autoimmune hepatitis (AIH), although the serologic profiles typically differ. ICI-induced liver injury predominantly involves CD3+ and CD8+ T lymphocytes, similar to other immune-mediated processes, although it lacks serologic markers such as elevated antinuclear antibody and IgG, and centrilobular zonal necrosis is uncommon, suggesting less zone-selective hepatocyte necrosis [3]. Management of ICI-induced hepatitis generally does not require systemic corticosteroids for less severe cases, and histologic analysis can help guide treatment [4].

Here, we describe a case of pembrolizumab-induced immune-mediated hepatitis that developed three months after the discontinuation of therapy. The patient required corticosteroid therapy and demonstrated histologic findings consistent with ICI-induced hepatitis with minimal histologic cholestasis on liver biopsy. Our case contributes to the growing recognition of delayed-onset hepatitis and underscores the need for ongoing monitoring for immune-mediated liver injury even after the cessation of immunotherapy.

## Case presentation

A 59-year-old female with a history of gallbladder adenocarcinoma presented with delayed-onset immune-mediated hepatitis following pembrolizumab therapy. Her past medical history was notable for gallbladder adenocarcinoma, hypertension, and a left internal carotid artery thrombus for which she underwent a decompressive left hemicraniectomy on August 23, 2024, complicated by deep venous thrombosis (DVT) and pulmonary embolism (PE) without right heart strain. Due to her recent neurosurgery, anticoagulation was initially withheld. During that admission, she underwent inferior vena cava (IVC) filter placement, tracheostomy, and placement of a percutaneous endoscopic gastrostomy (PEG) tube. She was ultimately discharged to a skilled nursing facility on apixaban on September 14, 2024.

**Figure 1:**
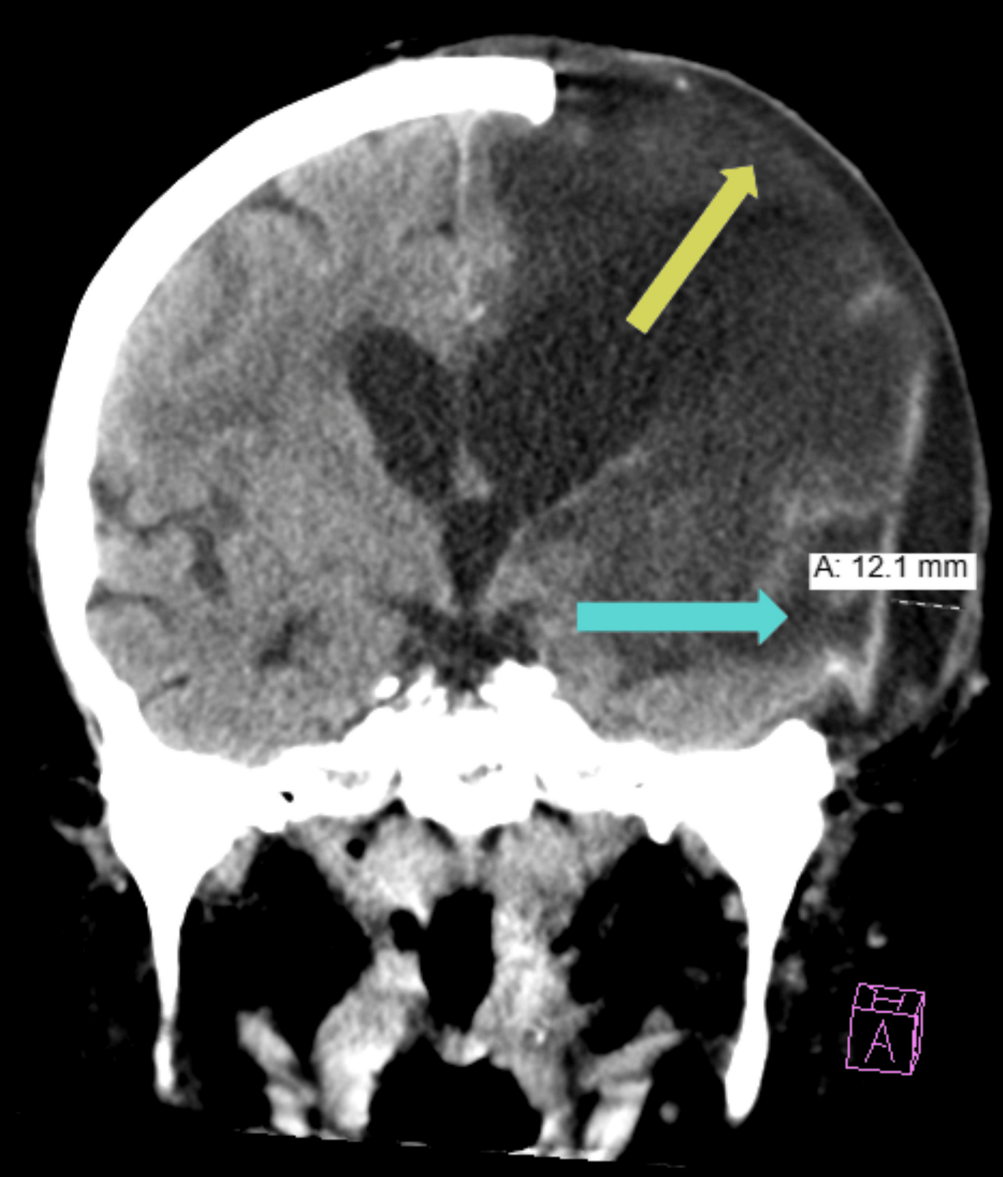
Noncontrast CT Head Redemonstrated postoperative changes of decompressive left hemicraniectomy with persistent mass effect and transcalvarial brain herniation (yellow arrow), similar to the prior examination. There is an interval increase in the size of the hypodense extra-axial fluid collection (blue arrow) subjacent to the craniectomy site, measuring up to 12 mm in greatest thickness, previously measured at 5 mm on MRI brain dated September 17, 2024. No significant midline shift.

**Figure 2:**
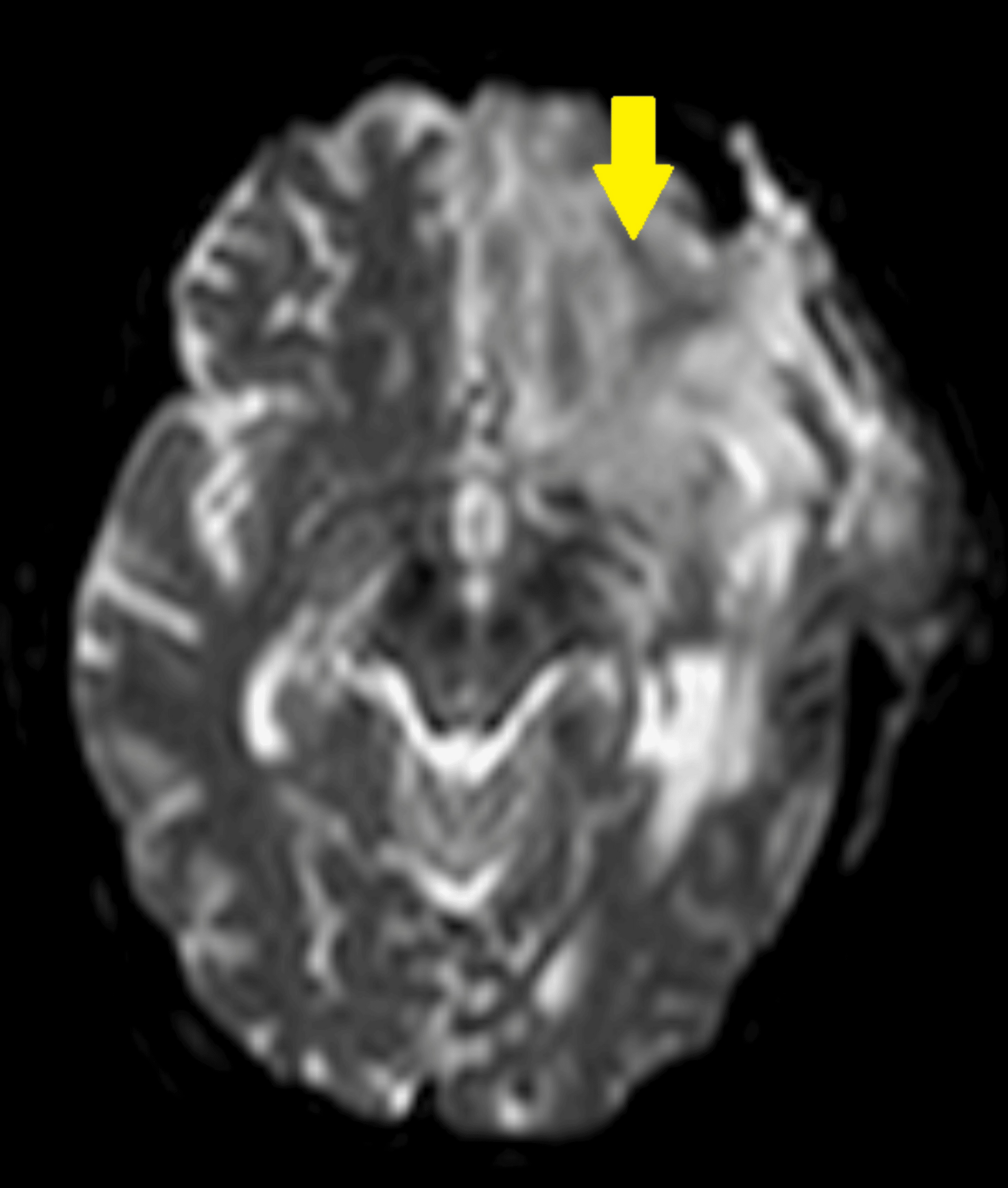
Axial DWI MRI Brain Without Contrast Image quality is limited by patient movement, and the study ended early after the patient was unable to tolerate lying supine for the entire duration of image capture. Although image interpretation is limited by the resolution, it demonstrates evolving late subacute infarction in the left anterior and middle cerebral artery distributions (yellow arrow). DWI: diffusion-weighted imaging.

Later that same day, she presented to the emergency department with a new-onset facial droop, lethargy, and drowsiness. A CT scan of the head (Figure 1) showed no acute findings, and brain MRI revealed an evolving left subacute infarct within the prior infarct territory (Figure 2). She was managed conservatively with aspirin and atorvastatin 40 mg PO daily and admitted for management of hyponatremia and possible infection. 

Hyponatremia, with a free water deficit of 1.3 liters, was corrected using dextrose 5% solution and increased free water PEG flushes. A urinalysis suggested a possible infection, and empiric antibiotics (levofloxacin 750 mg IV every 24 hours for 6 days, followed by one dose of ceftriaxone) were initiated as urine cultures grew extended-spectrum beta-lactamase (ESBL) Klebsiella pneumoniae at 10,000-100,000 CFU/mL. The infectious disease physician determined this to be asymptomatic bacteriuria, so antibiotics were stopped after the first dose of ceftriaxone 1 g IV.

**Figure 3:**
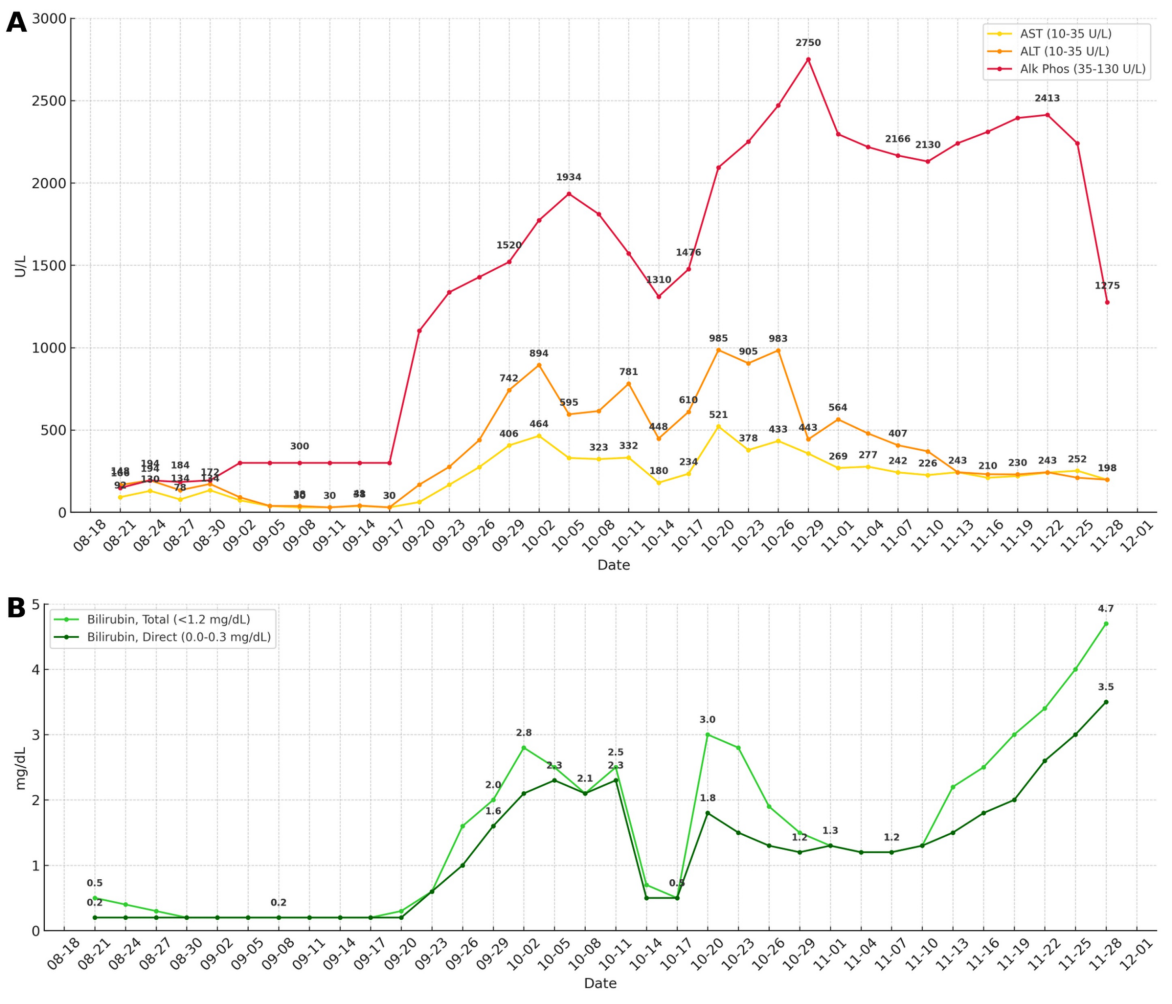
Patient's Laboratory Trends (A) Liver enzyme trends starting approximately three months prior to the patient’s admission and continuing through the hospitalization. The patient’s ALT and AST peaked at 985 U/L and 521 U/L, respectively, around October 17, 2024. Alkaline phosphatase peaked at 2,750 U/L on October 11, 2024. (B) Bilirubin trends over time beginning three months prior to admission. Total bilirubin peaked at 4.7 mg/dL on December 1, 2024, and direct bilirubin peaked at 3.5 mg/dL on November 30, 2024. The delayed bilirubin elevation relative to transaminase spikes suggests evolving ductal or cholestatic injury over time. However, this trend is confounded by the fact that her Lipitor had been held since admission for elevated transaminases from her baseline and was subsequently restarted as atorvastatin 20 mg (home dose 40 mg) on September 7, 2024, and continued at this dose for the duration of the hospital admission. Reference values for lower and upper limits of the normal range are included in the figure legend.

**Figure 4:**
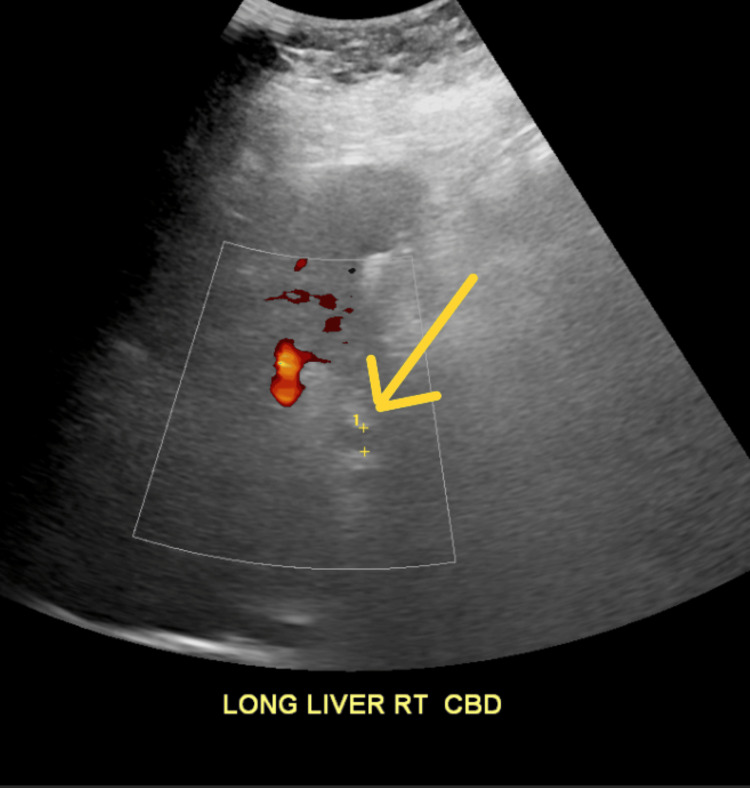
RUQ Abdominal Ultrasound of Liver Ultrasound of the abdomen showed increased echogenicity with nodular liver surface. The gallbladder is absent, consistent with prior cholecystectomy. The common bile duct measures 7 mm (yellow arrow). RUQ: right upper quadrant.

On September 23, 2024, liver function tests (LFTs) began rising significantly, including aspartate aminotransferase (AST), alanine aminotransferase (ALT), and alkaline phosphatase (ALP), while bilirubin initially remained stable (Figure 3). Abdominal ultrasound showed a cirrhotic-appearing liver without ductal dilatation, consistent with prior cholecystectomy (Figure 4). Tumor markers, including carcinoembryonic antigen (CEA) and carbohydrate antigen 19-9 (CA 19-9), were significantly elevated. 

**Figure 5:**
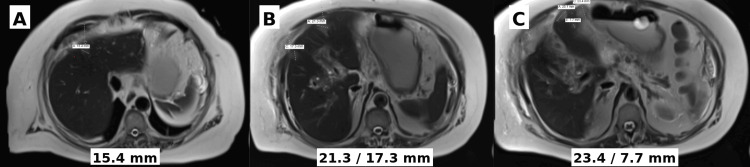
Axial T2 Weighted MRI Abdomen Axial T2-weighted MRI of the abdomen showing multiple hyperintense lesions concerning for liver metastases. Arrows and measurements were added in the imaging software by the radiologist and cannot be modified or removed; larger font is included in each corresponding image. (A) A 1.5 cm lesion in segment 4A of the liver; (B) a 1.7 cm lesion in segment 8 of the liver and a 2.1 cm lesion in segment 2/3 of the liver; (C) a cluster of three lesions in liver segment 3 measuring 2.3 cm, 2.3 cm, and 0.8 cm.

To further evaluate a new questionable 9-mm lesion seen on ultrasound and clarify the etiology of hepatic dysfunction, an MRI of the liver was obtained on September 30, 2024, once she was able to tolerate being supine for the study. Although limited by motion artifact, the study demonstrated diffuse periportal edema and at least six T1 hypointense, mildly T2 hyperintense lesions with restricted diffusion and peripheral enhancement, consistent with metastatic disease. No significant bile duct dilatation or abdominal lymphadenopathy was observed. Notable MRI findings are shown in Figure 5. 

**Figure 6:**
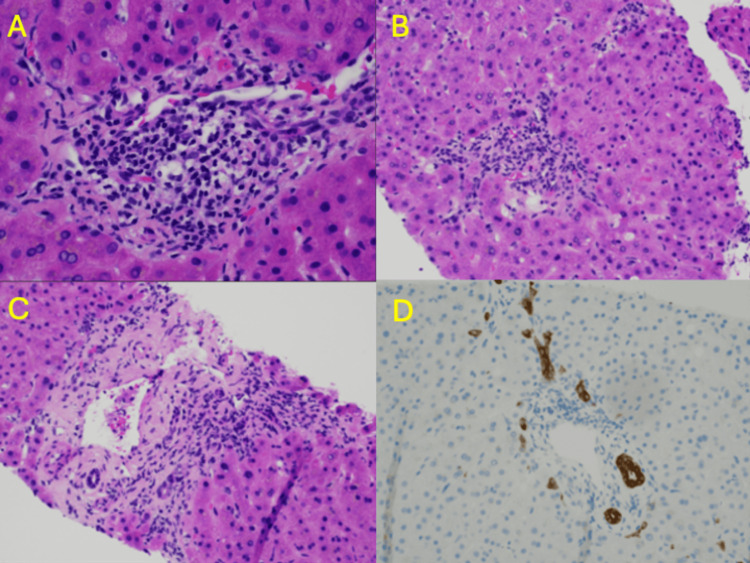
Histology Slides (A) Portal inflammation with glycogenated nuclei in periportal hepatocytes, 40× magnification; (B) minimal lobular activity (right upper corner of image) and cholestasis, 20× magnification; (C) occasional bile duct injury, 20× magnification; (D) CK7 immunostain, 20× magnification.

Due to worsening transaminases and diagnostic uncertainty, a liver biopsy was performed on September 27, 2024. The hematoxylin and eosin (H&E) sections showed mild infiltration of most portal tracts by mixed types of inflammatory cells, including lymphocytes, plasma cells, and rare neutrophils and eosinophils. The background liver showed frequent glycogenated nuclei, suggesting underlying metabolic syndrome or diabetes mellitus (Figure 6A). The periportal and lobular activities were minimal (Figure 6B). Occasional bile duct injury was noted, featuring degenerative appearance of cholangiocytes displaying eosinophilic cytoplasm and unevenly spaced nuclei, but distinct bile duct loss or ductopenia was not observed (Figure 6C). CK7 immunostain marked native bile ducts and mild ductular reaction (Figure 6D). Trichrome stain showed minimal perisinusoidal fibrosis, iron stain did not show a significant increase in iron deposition, reticulum stain demonstrated normal hepatic plate thickness, and no alpha-1 antitrypsin globules were identified on PAS-D stain (not shown). The overall histomorphologic features were compatible with a hepatocytic injury pattern. The differential diagnosis included drug-induced liver injury, infection, and systemic conditions [5]. Given the patient’s history of ICI therapy, the pathologic changes present on this liver biopsy specimen were compatible with immune checkpoint inhibitor therapy-related liver toxicity. 

Alternative diagnoses considered were statin-induced drug injury, autoimmune hepatitis, shock liver, metabolic syndrome, Wilson’s disease, MASLD, alcoholic hepatitis, and viral hepatitis. Peripheral eosinophilia was absent, which makes statin-induced drug injury less likely, although it is not a sensitive marker. Autoimmune serologies, including ANA, ASMA, AMA, and IgG, were all negative or normal. An extensive infectious workup, including viral hepatitis serologies, cytomegalovirus (CMV), and Epstein-Barr virus (EBV) testing, was unrevealing. Her last recorded hemoglobin A1c in August 2024 was 6.8%.

Review of her oncologic history documented by the admitting team revealed that she had undergone a laparoscopic cholecystectomy in April 2023 for presumed benign disease, during which incidental T2N0 gallbladder adenocarcinoma was diagnosed. Initial imaging did not reveal metastatic disease. However, a follow-up diagnostic laparoscopy in July 2023 identified poorly differentiated carcinoma with neuroendocrine features, and six of nine lymph nodes were positive for malignancy. Immunohistochemistry favored gallbladder origin (CK7+, CDX2+, CK20−). The patient was initially treated with chemotherapy (regimen unknown) before transitioning to pembrolizumab, with her final dose administered in June 2024. Due to a lack of electronic health record interoperability and the patient’s condition, direct records from her primary oncologist were not accessible. However, information regarding her cancer staging and treatment was verbally confirmed by the treating hospitalist during hospitalization.

Based on the clinical course, biopsy findings, and exclusion of infectious or autoimmune etiologies, the patient was diagnosed with pembrolizumab-induced immune-mediated hepatitis by the hematologist. The patient was initially started on IV methylprednisolone 100 mg daily on October 2, 2024. This was reduced to 80 mg IV (1 mg/kg) daily on October 8 for two days, with plans to discontinue therapy given uncertainty in etiology if the treatment failed to show benefit. However, due to a rebound in LFTs, she was restarted on oral prednisone 40 mg daily with a prolonged taper. Her LFTs began trending downward following steroid reinitiation, demonstrating a temporal association between corticosteroid therapy and transaminase improvement.

Although metastases were present, they were stable. The rise in LFTs without radiographic progression and histology favored an immune etiology. Unfortunately, due to fragmented records, her palliative course and cause of death were not documented, although it was confirmed with her primary oncologist that the patient had passed away. Consent for de-identified case publication was granted by the patient’s spouse prior to discharge.

## Discussion

Unlike many prior reports of delayed-onset ICI hepatitis that relied primarily on clinical and biochemical features without histologic confirmation, this case included a liver biopsy most compatible with ICI-induced hepatitis, adding more diagnostic clarity. ICIs like pembrolizumab have transformed oncology but carry a risk of irAEs, including immune-mediated hepatitis, which affects 5-10% of patients treated with ICIs [6]. Although most cases occur within the first 6-14 weeks of therapy, delayed-onset hepatitis, sometimes months after cessation, is increasingly recognized [7]. Our patient’s injury occurred long after completing pembrolizumab, emphasizing the need for prolonged monitoring.

Liver cancer patients may be particularly susceptible to immune damage. A meta-analysis showed that ICI hepatotoxicity caused a roughly 13-14% rise in AST and ALT in those with primary liver cancer, compared to about 5% in other malignancies [8]. PD-1 blockade disrupts immune tolerance, allowing cytotoxic T-cell activation that may damage hepatocytes or bile ducts [9]. Unlike AIH, ICI-induced hepatitis usually lacks autoantibodies or hypergammaglobulinemia [10]. Liver biopsy remains essential to distinguish this entity from other causes.

Histologically, immune-mediated hepatitis presents as lobular hepatitis with mild portal inflammation and minimal plasma cells. Zhang et al. described eight cases of anti-PD-1 liver injury, mostly showing acute lobular hepatitis. One had steatohepatitis, another cholestasis, but none displayed classic AIH histology [11]. While uncommon, cholangiopathy due to PD-1 inhibitors has been documented. Stuart et al. described a case progressing from hepatitis to sclerosing cholangitis, with persistent bile duct injury despite immunosuppression and ursodeoxycholic acid (UDCA) [12]. A similar case by Yang et al. involved severe cholestatic hepatitis post-pembrolizumab without biliary obstruction, reinforcing the variability and latency of irAEs [7].

Generally, grade 1 toxicities are managed with close monitoring, with withdrawal of ICI starting for most grade 2 toxicities. Grade 3 or 4 toxicities generally warrant suspension of the agent as well as initiation of high-dose corticosteroids [13]. Our patient’s initial partial steroid response prompted consideration of mycophenolate mofetil (MMF), a commonly used second-line agent [14]. Hwang et al. found that about 16% of ICI hepatitis cases are steroid refractory, with MMF effective in the majority [15]. Tacrolimus or azathioprine may be considered if MMF fails. Infliximab is avoided due to hepatotoxicity risks. Our patient eventually responded to steroids without the need for MMF, given the patient’s poor baseline functional status and likely inability to tolerate the additional medication. According to the admission documentation, the Keytruda was supposed to be ongoing therapy and was held after her CVA and was supposed to be resumed pending improvement in her functional status. When considering her future treatment, rechallenging after experiencing ICI hepatitis carries a significant recurrence risk, and given our patient’s grade 4 toxicity, pembrolizumab was discontinued permanently.

It was challenging to determine if the new liver metastases were contributing to a mixed etiology of the transaminase rise. However, the dramatic uptrend in transaminases in the absence of a concurrent bilirubin rise occurred despite stable metastatic disease burden on admission, suggesting that the hepatic dysfunction was more likely immune-mediated rather than tumor-related. In November, the patient’s cancer ultimately progressed very rapidly, and the patient passed from cancer-related failure to thrive soon thereafter before the full effects of the steroids could be assessed. One major limitation of this report was limited access to the patient’s outside oncologic history, with a provider not in the network. The only documentation available was from what was discussed over the phone during multiple calls made during the hospital admission. Additionally, the patient passed before we could obtain a release for her previous oncologic records. The spouse agreed to publish her de-identified information prior to hospital discharge, and this was documented as such. This, however, gave us limited ability to access what prior chemotherapy regimens she received, the dose of pembrolizumab, and the length of exposure or number of chemotherapy cycles. The information published above was obtained from our chart review and verbal information provided by the patient’s primary oncologist during the acute hospitalization over the phone.

Another potential limitation was that our patient was on two potential agents that are rarely known to cause drug-induced liver injury (DILI). Atorvastatin was restarted during the admission, close to the uptrend in transaminases. Although it was at a lower dose than her previous regimen, the reaction to statins is idiosyncratic and can occur at any point during administration. Levofloxacin was also used to treat her UTI, which, although rare, can also be associated with DILI. The biopsy was most consistent with injury caused by ICIs.

## Conclusions

This case highlights immune-mediated hepatitis as a potential delayed complication of pembrolizumab, occurring even months after discontinuation. The patient’s complex presentation illustrates how immune-related adverse events can mimic disease progression or infection, underscoring the need for a broad differential. Histology revealed a CD8+ T-cell-predominant pattern consistent with ICI-induced hepatitis, despite negative autoimmune serologies. While her liver function improved with corticosteroids, she ultimately passed from her underlying malignancy, reflecting the challenges of managing irAEs with complex comorbidities in the setting of fragmented health records. Rising transaminases in cancer patients should prompt consideration of irAEs, and continued case reporting is essential for improving recognition and management.
